# Postoperative dysphagia caused by a delay in mandibular fracture treatment in a patient with severe intellectual disability: a case report

**DOI:** 10.1186/s13256-021-03116-6

**Published:** 2022-01-03

**Authors:** Shinsuke Yamamoto, Masanori Nashi, Keigo Maeda, Naoki Taniike, Toshihiko Takenobu

**Affiliations:** Department of Oral and Maxillofacial Surgery, Kobe City Medical Center General Hospital, 2-1-1 Minatojima Minamimachi, Chuo-ku, Kobe, Hyogo 650-0047 Japan

**Keywords:** Mandibular fracture, Severe intellectual disability, Postoperative dysphagia, Dysphagia rehabilitation

## Abstract

**Background:**

The postoperative complications of mandibular fracture include malocclusion, infection, nonunion, osteomyelitis, and sensorial mental nerve dysfunction. However, there are no reports regarding postoperative dysphagia as a complication of mandibular fracture. Herein, we report a rare case of postoperative dysphagia caused by delayed mandibular fracture treatment in a patient with severe intellectual disability.

**Case presentation:**

A 46-year-old Japanese male patient with severe intellectual disability fell down and struck his chin. The patient was referred to our department 10 days after the accident. Upon examination, he could not close his mouth because of severe left mandibular body fracture. Open reduction and internal fixation was performed under general anesthesia 16 days after sustaining the injury, and normal occlusion was eventually achieved. However, the patient could not swallow well a day after surgery. He was then diagnosed with postoperative dysphagia caused by disuse atrophy of muscles for swallowing based on videoendoscopic examination findings. Adequate dysphagia rehabilitation could not be facilitated because of the patient’s mental status. Postoperative dysphagia did not improve 21 days after surgery. Therefore, percutaneous endoscopic gastrostomy was required.

**Conclusions:**

The treatment course of the patient had two important implications. First, postoperative dysphagia caused by disuse atrophy may occur if treatment is delayed in severe mandibular body fracture. Second, in particular, if a patient with severe intellectual disability develops postoperative dysphagia caused by disuse atrophy, adequate dysphagia rehabilitation cannot be facilitated, and percutaneous endoscopic gastrostomy may be required. Therefore, early open reduction and internal fixation is required for mandibular fracture in a patient with severe intellectual disability.

## Background

People with severe intellectual disability have communication difficulties, and medical treatments for these individuals are limited [1]. Moreover, owing to their physical and psychological characteristics, they can sometimes experience different complications during the perioperative period [1, 2]. By contrast, if appropriate treatment is not immediately provided after sustaining facial bone fracture, the injury often malunites, and soft tissues shrink and contract. All of these factors can delay treatment and can cause malocclusions and facial deformity [3, 4]. Herein, we report a rare case of postoperative dysphagia caused by a delay in severe mandibular body fracture treatment in a patient with severe intellectual disability. Moreover, the importance of early open reduction and internal fixation (ORIF) is discussed.

## Case presentation

**Fig. 1:**
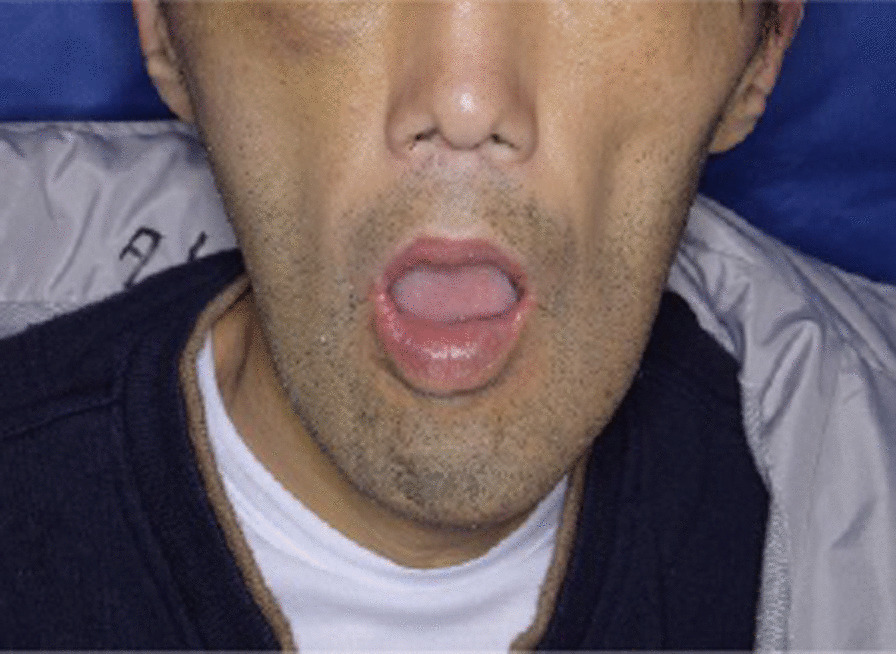
Facial image obtained during the initial visit showing that the patient could not close his mouth

**Fig. 2:**
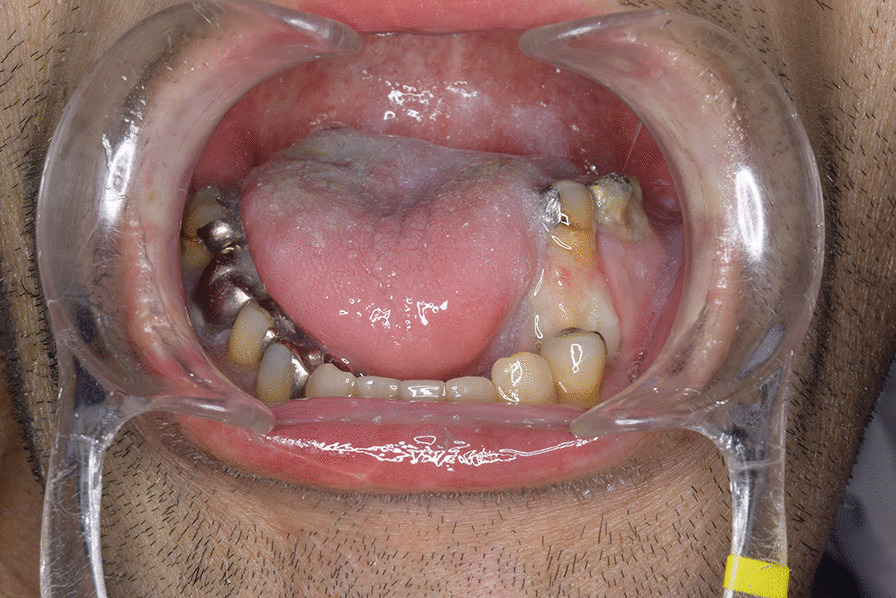
Intraoral image obtained during the initial visit showing anterior open bite and a significant gap between the left lower first and second premolars

**Fig. 3:**
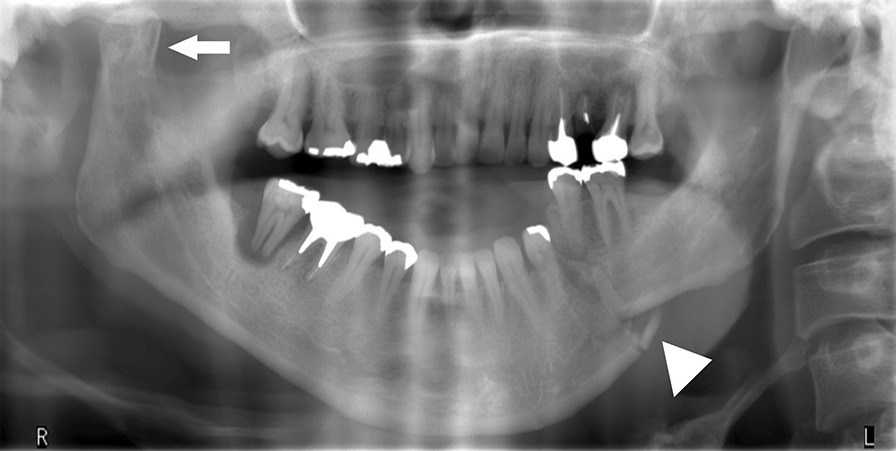
Panoramic image obtained during the initial visit showing left mandibular body (white arrowhead) and right condylar head (white arrow) fracture

**Fig. 4:**
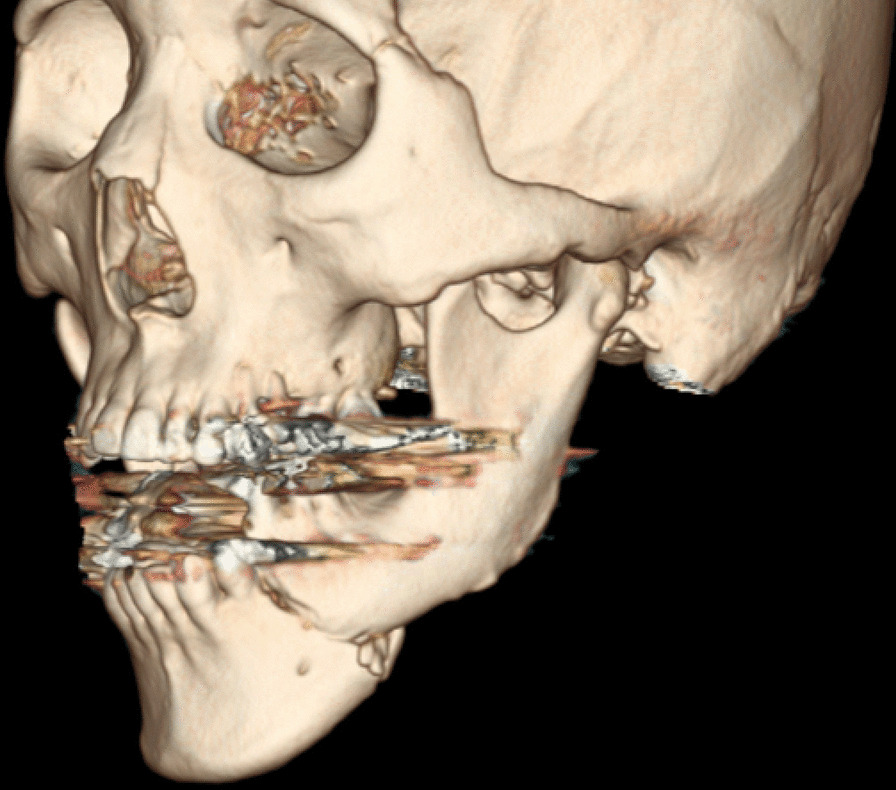
Three-dimensional computed tomography findings obtained during the initial visit showed fracture characterized by severe deviation of the left mandibular body

**Fig. 5:**
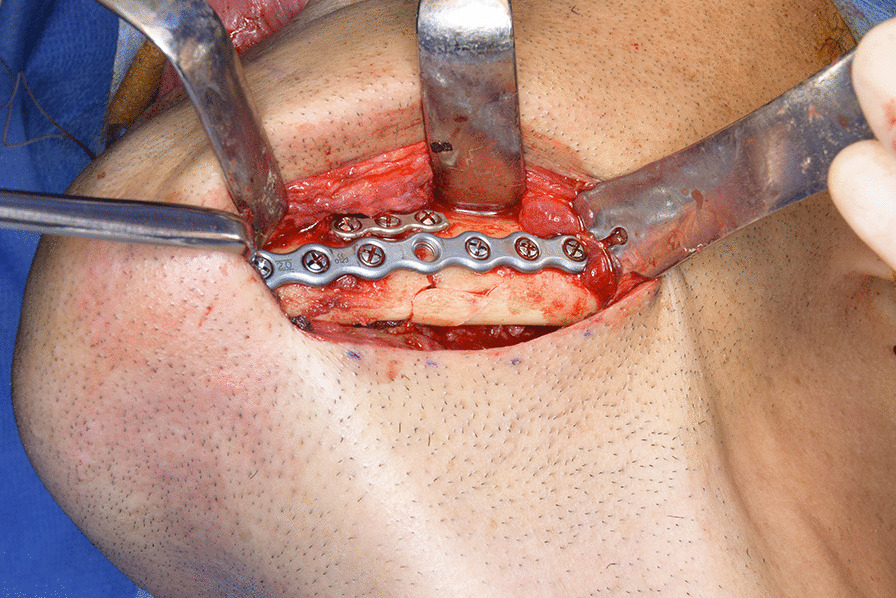
Intraoperative image showing that ORIF for left mandibular body fracture using a submandibular approach was performed

**Fig. 6:**
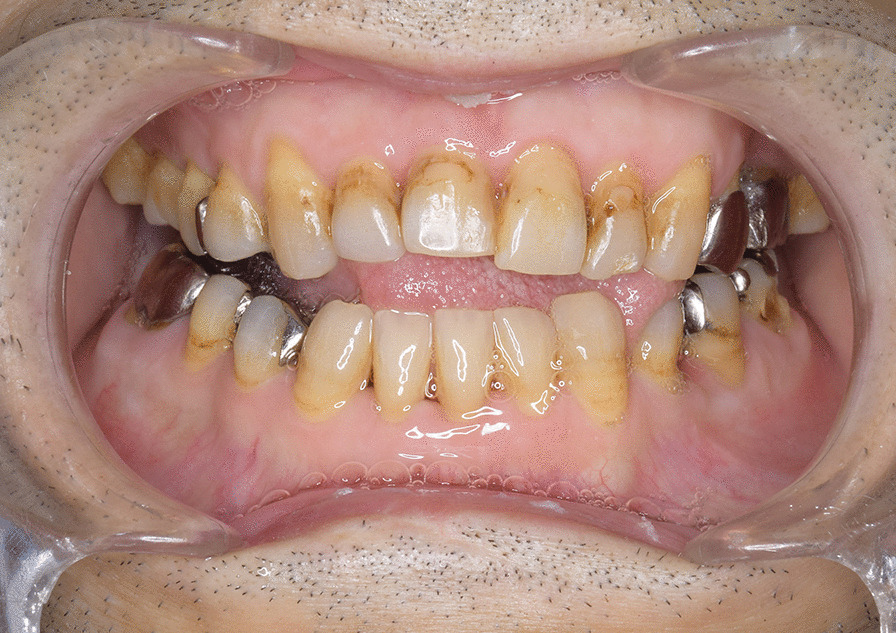
Intraoral image obtained after ORIF showing that normal occlusion was achieved

**Fig. 7:**
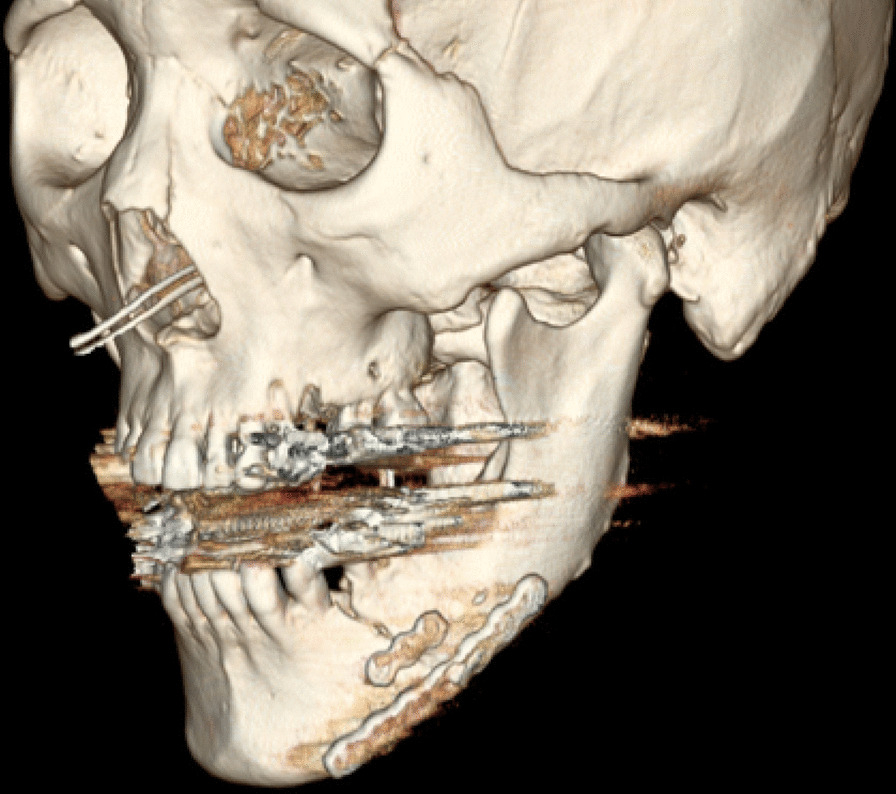
Three-dimensional computed tomographic findings obtained after ORIF showing that adequate reduction and fixation were obtained

**Fig. 8:**
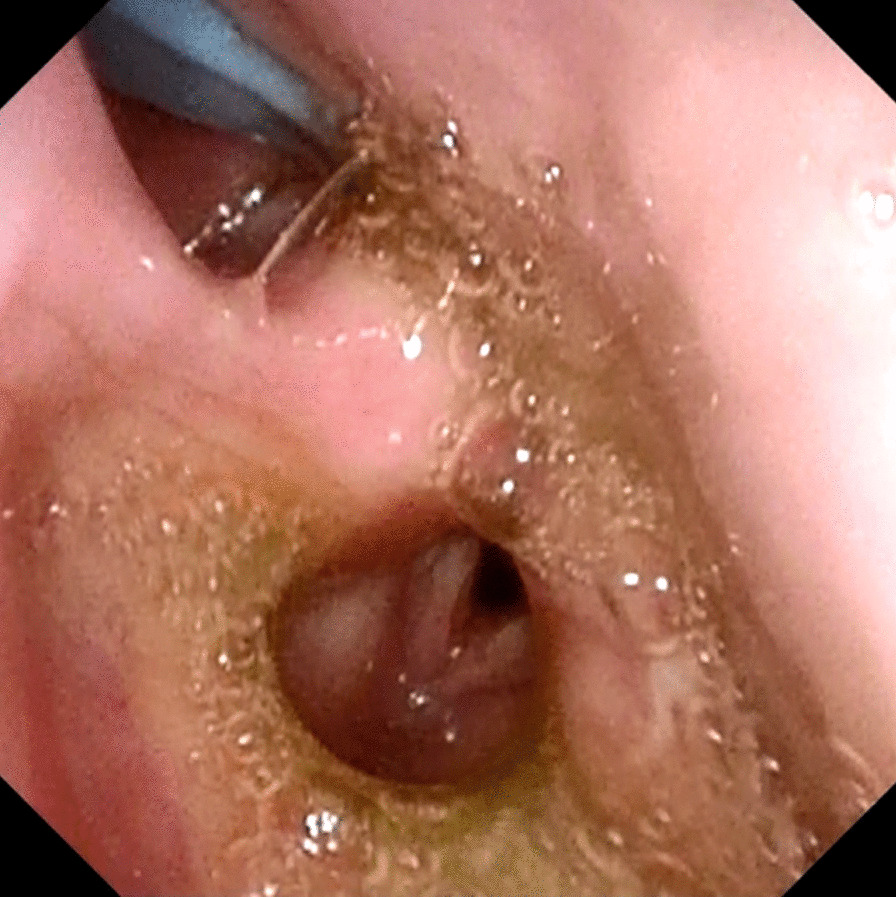
Findings of videoendoscopic examination of swallowing performed 21 days after surgery revealing significant saliva and jelly retention at the epiglottic vallecula and piriform fossa

A 46-year-old Japanese male patient with a medical history of severe intellectual disability and epilepsy was admitted to a support facility for persons with disabilities. The patient fell down and struck his chin in January 2020. Even before the injury, the patient had problems with verbal communication. He was self-reliant in performing activities of daily living and experienced no swallowing difficulty before the injury. He had been consuming normal food before the injury. He received treatment with carbamazepine 800 mg/day, risperidone 6 mg/day, chlorpromazine phenolphthalinate 300 mg/day, lorazepam 3 mg/day, sodium valproate 1000 mg/day, magnesium oxide 1000 mg/day, and flunitrazepam 2 mg/day. He was initially referred to another hospital and was diagnosed with mandibular fracture. Hence, open reduction and internal fixation (ORIF) was considered. However, the patient should be treated first at a psychopathic ward. Next, he was referred to another university hospital because of a significant risk of self-mutilation. However, the psychiatric ward was full. Finally, he was referred to our department and was accompanied by his mother and facility staff, 10 days after sustaining the injury. Clinical examination at the initial visit revealed productive cough and sialorrhea. In addition, there was conspicuous oral contamination with phlegm. The patient could not close his mouth because of anterior open bite and a significant gap between the left lower first and second premolars (Figs. 1 and 2). Panoramic radiography and computed tomography (CT) scan revealed left mandibular body and right condylar head fractures (Figs. 3 and 4). Moreover, chest radiography and CT scan showed infiltrative shadow in the lower lobe of both lungs. Blood tests had the following results: white blood cell count of 15,400/μL (normal range 3900–9800/μL) and C-reactive protein level of 14.74 mg/dL (normal range 0.00–0.50 mg/dL). He was diagnosed with mandibular fracture and aspiration pneumonitis and was admitted to the medical psychiatric unit (MPU) of our hospital. He had eaten liquid food before admission to our hospital, and a feeding tube was inserted at the time of admission to ORIF. After receiving ampicillin/sulbactam 12 g/day for 5 days for aspiration pneumonitis, ORIF using the submandibular approach for left mandibular body fracture was performed under general anesthesia, 16 days after sustaining the injury. Then, normal occlusion was achieved (Figs. 5, 6, and 7). The patient received conservative treatment for right condylar head fracture. Although oral intake was resumed a day after surgery, he could not properly swallow a jelly, and enteral feeding via a nasogastric tube was again required. Videoendoscopic examination of swallowing was performed by a nutrition support team 2 days after surgery. Results showed significant saliva retention at the epiglottic vallecula and piriform fossa (Fig. 8). Moreover, delayed elicitation of pharyngeal swallowing and poor transfer from the oral cavity to the pharynx were observed. The jelly was transferred from the oral cavity to the pharynx by gravity. He was then diagnosed with postoperative dysphagia owing to disuse atrophy of muscles for swallowing. Although oral care, thermal–tactile stimulation, and cervical range of motion training were continued, adequate dysphagia rehabilitation, including direct and indirect training, could not be facilitated. Re-evaluation of swallowing function was performed 21 days after surgery. However, there was no improvement in postoperative dysphagia. Practically, test of swallowing thickened water as a direct training was performed. Choking and wet hoarseness were not observed. However, an abnormal swallowing sound was heard, and most of the jelly was aspirated from the pharynx after swallowing. Therefore, percutaneous endoscopic gastrostomy was performed 23 days after surgery. Then, the patient was transferred to the original support facility for persons with disabilities 52 days after surgery. Medical restraint was recommended by psychiatrists and the MPU staff to prevent heavy self-mutilation and harming medical staff during hospitalization according to the Act on Mental Health and Welfare for the Mentally Disabled (Japanese Mental Health Law).

## Discussion

To the best of our knowledge, this is the first described report of a case of postoperative dysphagia caused by a delay in mandibular fracture treatment. The findings in this case have two important implications. First, postoperative dysphagia caused by disuse atrophy may occur if treatment is delayed in severe mandibular fracture. The postoperative complications of mandibular fracture include malocclusion, infection, nonunion, osteomyelitis, and sensorial mental nerve dysfunction [5–8]. Previous studies did not show any significant correlation between complications arising during treatment of mandibular fracture [6, 8]. Patient’s comfort and well-being, as well as any psychological effects caused by disfigurement of unrepaired mandibular fractures, should be considered with a reasonable length of treatment delay [8]. In fact, most patients received treatment within 2–4 days after injury [6]. In the study of Patrocinio *et al*. [5], about 73% of patients were treated within the first week, and 14% were managed after 15 days or more. In this case, ORIF was performed 16 days after the accident because of difficulties in securing a psychiatric bed and treatment for aspiration pneumonia prior to ORIF. Ohba *et al*. [9] reported a case of aspiration pneumonia accompanied by impaired swallowing function owing to a double mandibular fracture. The symptoms of aspiration pneumonia improved immediately after surgery. Hence, open reduction should be performed immediately to prevent aspiration pneumonia caused by swallowing dysfunction. In this case, long-term open bite attributed to severe mandibular fracture caused sialorrhea and aspiration pneumonia via decreased swallowing function. Interestingly, Shimizu *et al*. [10] assessed the geniohyoid muscle via ultrasonography perioperatively in patients who underwent thoracotomy and laparotomy. Results showed that muscle atrophy due to surgical invasion or disuse may occur in the deglutition muscles, as in the limb muscles. Furthermore, muscle atrophy can develop in the early postoperative period and can persist even after 2 weeks from surgery. In the case of symphyseal, parasymphyseal, or condyle fracture, deviation of the mandible is mild because both side mastication muscles are balanced. On the other hand, in the case of mandibular body or angle fracture, the masseter, medial pterygoid, and temporalis muscles contribute to the superior and medial displacement of the proximal segment. Furthermore, digastric, mylohyoid, and geniohyoid muscles contribute to the inferior displacement of the distal segment. Therefore, there is higher risk of atrophic dysphagia by the local neuromuscular misbalance owing to significant gap between proximal and distal segments. In this case, normal occlusion was achieved via ORIF, and severe mandibular fracture (that is, structural disorder) was managed. Nevertheless, disuse atrophy (that is, functional disorder) occurred during the time between falling and ORIF, and dysphagia was still observed after surgery. The reserved capacity is reduced among elderly individuals and those with severe intellectual disability [11, 12]. Thus, not only structural disorder but also functional disorder should be considered in planning the treatment for severe deviated mandibular fracture.

Second, patients with severe intellectual disability can develop postoperative dysphagia caused by disuse atrophy. Hence, adequate dysphagia rehabilitation cannot be facilitated, and percutaneous endoscopic gastrostomy may be required. Therefore, early ORIF is recommended for mandibular fracture in a patient with severe intellectual disability. In addition to videoendoscopic examination findings, on the basis of the facts that satisfactory occlusion was achieved with ORIF, the patient was on a soft diet and could feed without assistance before the accident, the dosage and type of medications were not changed, and severe anterior open bite persisted for more than 2 weeks, the patient was diagnosed with disuse atrophy of muscles for swallowing, including the tongue. Normally, the combination of compensatory techniques (that is, postural maneuvers), indirect therapy (exercises that can strengthen swallowing muscles, including oral care), and direct therapy (exercises for swallowing) was required for dysphagia rehabilitation [13–15]. However, we could not adequately facilitate dysphagia rehabilitation. Thus, only oral care, thermal–tactile stimulation, and cervical range of motion training were continuously provided as indirect therapy and percutaneous endoscopic gastrostomy was required in this case. Therefore, we should keep in mind that “people with intellectual disabilities vary in characteristics, abilities, and preferences, but health professionals must not hold back from treating them” [1]. Thus, ORIF should be immediately performed. Moreover, efforts should be taken to prevent severe complication in individuals with severe maxillofacial injuries.

This case report had limitations. That is, we cannot completely rule out the risk of drug-induced dysphagia [15–17] because the patient was under treatment with several antiepileptic and antipsychotic drugs. However, the dose was not changed before and after surgery. Furthermore, we did not evaluate swallowing function preoperatively or reevaluate after a long period after surgery to decide whether this complication is related to the delayed surgery or disability of the patient [18, 19]. Thus, further studies on cases of postoperative dysphagia in patients with mandibular fracture should be conducted to validate whether delay in mandibular fracture treatment can cause dysphagia.

## Conclusions

The treatment course of the patient had two important implications. First, postoperative dysphagia caused by disuse atrophy may occur if treatment is delayed in severe mandibular body fracture. Second, in particular, if a patient with severe intellectual disability develops postoperative dysphagia caused by disuse atrophy, adequate dysphagia rehabilitation cannot be facilitated, and PEG may be required. Therefore, early ORIF is required for mandibular fracture in a patient with severe intellectual disability.

## Data Availability

Data sharing is not applicable to this article as no datasets were generated or analyzed during the current study.

## Abbreviations

ORIF: Open reduction and internal fixation
PEG: Percutaneous endoscopic gastrostomy
CT: Computed tomography
MPU: Medical psychiatric unit
